# Acute toxicity of vipoxin and its components: is the acidic component an “inhibitor” of PLA_2_ toxicity?

**DOI:** 10.2478/v10102-012-0028-z

**Published:** 2012-12

**Authors:** Vasil N. Atanasov, Silviya Stoykova, Yana Goranova, Mariana Mitewa, Svetla Petrova

**Affiliations:** 1 Sofia University “St. Kl. Ohridski”, Faculty of Chemistry and Pharmacy, Department of Analytical Chemistry, Laboratory of Biocoordination and Bioanalytical Chemitsry, Sofia, Bulgaria; 2 Military Medical Academy, Emergency Toxicology Clinic, Sofia, Bulgaria; 3 Sofia University “St. Kl. Ohridski”, Faculty of Biology, Department of Biochemistry, Laboratory of Enzymology, Sofia, Bulgaria

**Keywords:** vipoxin, phospholipase A_2_, acidic component, acute toxicity

## Abstract

Vipoxin is a heterodimeric neurotoxin isolated from the venom of the Bulgarian long-nosed viper *Vipera ammodytes meridionalis*. Vipoxin represents a noncovalent association of two subunits – a basic and toxic phospholipase A_2_ enzyme, and an acidic non-enzymatic component (vipoxin's acidic component). It was postulated that the phospholipase A_2_ subunit was more toxic than the whole vipoxin complex and the function of the acidic component was to reduce the enzymatic and toxic activities of the basic phospholipase A_2_. In the present study, we report new data on the acute toxicity (LD_50_) of vipoxin and its individual separated components. Vipoxin LD_50_ (mice, i.p. and i.v.) values were found to be 0.7–1.2 mg/kg b.w. (i.p.) and 0.9–1.3 mg/kg b.w. (i.v.). The established LD_50_ values for the separated pure phospholipase A_2_ subunit are higher – 10.0–13.0 mg/kg b.w (i.p.) and 2.2–3.0 mg/kg b.w. (i.v.), *i.e.* the individual phospholipase A_2_ subunit displays less toxic activity than vipoxin, contrary to the data published in the literature. The reconstituted vipoxin complex (obtained after preliminary incubation of pure separated phospholipase A_2_ and acidic component showed enzyme activity and toxicity comparable to that of the native vipoxin complex. Addition of acidic component to the phospholipase A_2_ subunit showed a positive effect on the enzymatic activity, reaching maximal enzyme reaction rate of acidic component to phospholipase A_2_ molar ratio of 0.8:1 on using 4-nitro-3-octanoyloxy-benzoic acid as substrate. For the first time we showed that the acidic subunit was absolutely required for the toxic activity of vipoxin. Based on the obtained results, we assume that the function of the acidic component is to stabilize the neurotoxin's quaternary structure, required for its toxic and enzymatic activities, similarly to the role of the acidic component of crotoxin.

## Introduction

Vipoxin is the main toxic component isolated from the venom of long-nosed viper living in in Bulgaria – *Vipera ammodytes meridionalis (V.a.m)*. Tomovic (Tomovic, 2006) identified it as *V. amm. montandoni* although the two subspecies are different. Other studies based on chromatographic analyses showed that the crude venoms of both viper subspecies contained vipoxin (Bardarov, 2002). Vipoxin is a complex of two subunits – a basic and toxic phospholipase A_2_ enzyme (PLA_2_) and an acidic non-enzymatic component (VAC). Both subunits contain 122 amino-acid residues, seven disulphide bridges and display 62% sequence identity (Mancheva *et al.*, 1987).

According to the published and accepted data, the separated PLA_2_ from vipoxin loses irreversibly its toxicity after 3–4 days and its enzymatic activity within two weeks (Aleksiev & Shipolini, 1973; Aleksiev & Tchorbanov, 1976). Reconstitution of the vipoxin complex by mixing the preliminarily separated and purified basic PLA_2_ and VAC (in 1:1 molar ratio) restored its properties and retained stability for years at 4 °C (Aleksiev & Tchorbanov, 1976).

VAC was originally characterized as “inhibitor” because of the measured lower enzymatic and toxic activities of the vipoxin complex compared to the separated pure PLA_2_ subunit measured at the same substrate concentration (Tchorbanov *et al.*, 1978).

The toxicity of isolated PLA_2_ (presented as LD_100_ value) was determined to be 1–3 µg per 20 g mouse after i.v. administration (Aleksiev and Shipolini, 1973) compared to the vipoxin LD_100_ toxicity value – 8 µg per 20 g mouse (Tchorbanov and Aleksiev, 1981). Actually, the toxicity presented in LD_100_ units is not correct and not very useful in toxicological studies and pharmacological testing. Our new studies on vipoxin intend to update the acute toxicity values and evaluate them in terms of the median lethal dose LD_50_.

## Materials and methods

Vipoxin and its components were isolated as described previously (Tchorbanov and Aleksiev, 1981; Atanasov *et al.*, 2009). Vipoxin was isolated from the crude venom by ion-exchange chromatography on SP-Sephades C-50 (Pharmacia, Sweden) using linear gradient from 0.05 M up to 0.4 M Tris-citrate buffer, pH 7.3. The vipoxin-containing fraction was dialyzed against water and lyophilized. The components of vipoxin were separated using FPLC on Mono S HR 5/5 column (Pharmacia, Sweden) equilibrated with 0.1 M acetate buffer (pH 4.0) in the presence of 6 M carbamide and eluted with a linear gradient of NaCl up to 0.5 M at a flow rate of 0.4 mL/min. The homogeneity of the enzyme was confirmed by SDS-PAGE.

### Toxicity tests

The acute toxicity was measured by the method of Prozorovsky (Prozorovsky *et al.*, 1978). Male ICR mice (18–25 g) were used in the experiments. The animals had access to food and water *ad libitum* and were maintained at 24 ± 2 °C with a 12 h light/dark cycle. Four experimental groups of two animals per group (dose) were used. All experiments and procedures with animals were approved and carried out under the control of the Ethical Committee on Animal Experiments of the Bulgarian Academy of Sciences. Vipoxin, PLA_2_, VAC and reconstituted vipoxin complex (a mixture of preliminarily separated and purified PLA_2_ and VAC in 1:1 molar ratio, incubated for up to 24 h at 4 °C) were diluted in saline and administered to the animals intraperitoneally (i.p.) or intravenously (i.v.). The mice were observed for 24 h. Dead animals were examined by gross autopsy. The experiments were performed in three independent repetitions.

### Enzyme assay

PLA_2_ enzyme activity was measured using 4-nitro-3-octanoyloxy-benzoic acid (NOBA) as substrate, according to the procedure of Holzer and Mackessy (1996). The standard assay mixture contained 220 µl of buffer (10 mM Tris-HCl, 5 mM CaCl_2_, 100 mM NaCl, pH 8.0), 20 µl of 1.5 mM NOBA in acetonitrile (synthesized according to Cho *et al.*, 1988) and 20 µl of appropriately diluted sPLA_2_ (about 0.2 µM) in the final volume of 260 µl. The enzyme activity (expressed as the initial velocity of the reaction) was calculated based on the increase in absorbance at 450 nm after 15 min. All assays were conducted in triplicate using Dynex (Dynex Technologies, USA) multiwell plate reader.

To analyze the effect of VAC on phospholipase A_2_ activity, a fixed molar concentration of the purified PLA_2_ subunit was pre-incubated with the same molar concentrations of VAC (1:1 molar ratio as in the native vipoxin). Incubation was carried out in 25 mM Tris-HCl buffer (pH 8.0) for 24 h at 4 °C. At different time intervals, aliquots from this mixture were withdrawn and the PLA_2_ activity was measured under the same conditions. The results were compared with the activity of individual pure PLA_2_ measured in the absence of VAC, as well as the activity of the original vipoxin complex, containing the same amount of PLA_2_.

## Results

The results on the acute toxicity of vipoxin and its individual subunits in mice are summarized in Table 1. The most intriguing result in our experiments was the higher toxicity of vipoxin compared to that of the isolated and purified PLA_2_. The LD_50_ values of vipoxin presented in mol/kg b.w. showed more than five times higher toxicity than that of PLA_2_ at i.v. administration and more than 20 times higher at i.p. route.

**Table 1:** Acute toxicity (LD_50_ values) and type of clinical signs induced by vipoxin, its components and reconstituted vipoxin.

Compound tested	Route of admin.	LD_50_ (mg/kg)	LD_50_ (µmol/kg)	Type of clinical signs
Vipoxin	i.p.	0.7-1.2	0.02–0.04	neurotoxic
Vipoxin	i.v.	0.9-1.3	0.03–0.04	neurotoxic
PLA_2_ subunit	i.p.	10–13	0.7–0.9	not specified
PLA_2_ subunit	i.v.	2.2-3.0	0.1–0.2	not specified
Acidic component	i.p.	>30	>2	no signs
Reconstituted vipoxin (PLA_2_ + VAC, 1:1)	i.p.	0.6-1.2	0.02–0.04	neurotoxic

Typical clinical signs of neurotoxic activity were observed when vipoxin was applied: agitation, ataxia, tremor and convulsions, paresis of hind limbs, seizure activity and usually death within 2–5 min after generalized seizure. The same toxic manifestations were noticed when the reconstituted vipoxin complex (PLA_2_:VAC in 1:1 molar ratio) was used. Surprisingly, in the case of the individual pure PLA_2_ subunit these signs were not observed. The animals were apathetic, with lack of neurotoxic features (only transitional paresis in isolated cases followed by complete animal recovery). Necropsies of the dead animals showed absence of any organ injuries, hemorrhages or other macroscopic changes.

We found that the enzymatic activity of the separated pure PLA_2_ subunit and the reconstituted VAC:PLA_2_ complex (in a 1:1 molar ratio, pre-incubated for different time intervals, followed by immediate activity assay), decreased more significantly in the case of individual PLA_2_ (by 50%) compared to the phospholipase A_2_ activity of the reconstituted complex (by 20%) (Table 2).

**Table 2:** Role of VAC on the stability and enzymatic activity of the toxic PLA_2_ subunit.

Pre-incubation time	Residual activity of separated PLA_2_ subunit (%)	Residual activity of reconstituted complex PLA_2_ + VAC (%)
1 min	100	100
5 min	100	120
10 min	100	115
20 min	100	114
30 min	99	114
40 min	98	113
50 min	98	108
24 h	63	78

Individual, pure PLA_2_ subunit and reconstituted complex PLA_2_ + VAC (1:1 molar ratio) were pre-incubated for different time intervals followed by immediate addition of NOBA and activity assay. The residual enzyme activity is expressed as the percentage of the initial velocity, measured immediately after the indicated time of preliminarily incubation without substrate.

## Discussion

Our results offer new insights into vipoxin toxicity and the role of its acidic component. For more than thirty years the vipoxin basic PLA_2_ subunit has been accepted and discussed as being more toxic (50–150 µg/kg b.w. (i.v.) than vipoxin complex (400 µg/kg b.w.) (Tchorbanov and Aleksiev, 1981). We revised these data and obtained completely new facts that necessitated a corrected interpretation. If our data are interpreted in units of mol/kg b.w. (not in mass units as in the previous studies), the toxicity of PLA_2_ (Mr 15 kDa) becomes 3.3–10 nmol/kg b.w. and that of vipoxin (Mr 30 kDa) – 13 nmol/kg b.w. These converted values revealed a comparable toxicity of vipoxin and individual PLA_2_ subunit.

Contrary to the previously published data, our experiments showed practically the same LD_50_ values for the reconstituted mixture of subunits and the original vipoxin (Aleksiev and Tchorbanov, 1976; Tchorbanov *et al.*, 1978) (Table 1). We established that the toxicity of vipoxin was practically the same using i.p. and i.v. administration routes whereas the i.v. administration of the individual PLA_2_ subunit led to a higher toxicity than did the i.p. one (Table 1). These findings support the presumption that the integrity of the whole complex is important for its toxic activity and raise questions about the VAC role on the penetration abilities complex charge and pI.

Our results showed that the toxicity of vipoxin and its PLA_2_ component did not fully correlate with the enzymatic activity. A possible explanation may be a differentiation between the two functions. According to the classification of Rosenberg (Rosenberg, 1997), the vipoxin PLA_2_ component should be classified as PLA_2_ displaying weak toxicity and high enzymatic activity. Alexiev and Tchorbanov (1976) established earlier that the stability of the reconstituted vipoxin increased whereas under the same conditions the enzymatic activity decreased.

According to our findings, VAC is crucial for vipoxin toxicity and VAC activity can not be designated as an “inhibitor” subunit. Georgieva *et al.* (2003) assumed that the main role of VAC was to stabilize the unstable PLA_2_ subunit. Furthermore, the structure-function relationship in vipoxin proposed a “chaperone” function of VAC, preventing unspecific binding of the PLA_2_ subunit. A similar synergistic action between the components has been already established for crotoxin, isolated from *Crotalus durissus terrificus* – a heterodimeric protein, composed of basic PLA_2_ (crotoxin basic, CB) and acidic non-enzymatic (crotapotin; crotoxin acidic, CA) subunits. It is considered that CA functions as a carrier of the CB subunit, reducing its non-specific interaction and leading to an enhanced toxicity of crotoxin (Bon and Jang, 1979; De'lot and Bon, 1993; Faure *et al.*, 1993; Rangel-Santos *et al.*, 2004).We think that vipoxin displays a similar structure-function relationship between its subunits. In this respect, the role of the vipoxin acidic component should be discussed as a factor stabilizing the complex structure and hence retaining its toxicity by preventing non-specific binding.

## Conclusions

In the study we report our preliminary data on acute toxicity testing of vipoxin and its components. As a result of the tests performed, we assume that the VAC subunit plays an important role for the toxic function of the whole complex. Although VAC is non-toxic by itself, its presence is absolutely needed for the toxicity of the whole toxic complex. Our new findings showed that the toxicity vipoxin was comparable to that of the individual toxic PLA_2_ subunit (µmol/kg b.w.), which is in contradistinction to the accepted understanding dated from the late 1970s. We also think that the acidic subunit is extremely important for the activity and stability of vipoxin.
